# FerrDb V2: update of the manually curated database of ferroptosis regulators and ferroptosis-disease associations

**DOI:** 10.1093/nar/gkac935

**Published:** 2022-10-28

**Authors:** Nan Zhou, Xiaoqing Yuan, Qingsong Du, Zhiyu Zhang, Xiaolei Shi, Jinku Bao, Yuping Ning, Li Peng

**Affiliations:** Research Institute, The Affiliated Brain Hospital of Guangzhou Medical University, Guangzhou 510370, China; Guangdong Engineering Technology Research Center for Translational Medicine of Mental Disorders, Guangzhou 510370, China; Guangdong Provincial Key Laboratory of Malignant Tumor Epigenetics and Gene Regulation, Guangdong-Hong Kong Joint Laboratory for RNA Medicine, Sun Yat-Sen Memorial Hospital, Sun Yat-Sen University, Guangzhou 510120, China; Breast Tumor Center, Sun Yat-Sen Memorial Hospital, Sun Yat-Sen University, Guangzhou 510120, China; College of Life Sciences, Sichuan University, Chengdu, 610064, China; College of Life Sciences, Sichuan University, Chengdu, 610064, China; Research Institute, The Affiliated Brain Hospital of Guangzhou Medical University, Guangzhou 510370, China; College of Life Sciences, Sichuan University, Chengdu, 610064, China; Research Institute, The Affiliated Brain Hospital of Guangzhou Medical University, Guangzhou 510370, China; Guangdong Engineering Technology Research Center for Translational Medicine of Mental Disorders, Guangzhou 510370, China; Guangdong Provincial Key Laboratory of Malignant Tumor Epigenetics and Gene Regulation, Guangdong-Hong Kong Joint Laboratory for RNA Medicine, Sun Yat-Sen Memorial Hospital, Sun Yat-Sen University, Guangzhou 510120, China; Medical Research Center, Sun Yat-Sen Memorial Hospital, Sun Yat-Sen University, Guangzhou 510120, China

## Abstract

Ferroptosis is a mode of regulated cell death characterized by iron-dependent accumulation of lipid peroxidation. It is closely linked to the pathophysiological processes in many diseases. Since our publication of the first ferroptosis database in 2020 (FerrDb V1), many new findings have been published. To keep up with the rapid progress in ferroptosis research and to provide timely and high-quality data, here we present the successor, FerrDb V2. It contains 1001 ferroptosis regulators and 143 ferroptosis-disease associations manually curated from 3288 articles. Specifically, there are 621 gene regulators, of which 264 are drivers, 238 are suppressors, 9 are markers, and 110 are unclassified genes; and there are 380 substance regulators, with 201 inducers and 179 inhibitors. Compared to FerrDb V1, curated articles increase by >300%, ferroptosis regulators increase by 175%, and ferroptosis-disease associations increase by 50.5%. Circular RNA and pseudogene are novel regulators in FerrDb V2, and the percentage of non-coding RNA increases from 7.3% to 13.6%. External gene-related data were integrated, enabling thought-provoking and gene-oriented analysis in FerrDb V2. In conclusion, FerrDb V2 will help to acquire deeper insights into ferroptosis. FerrDb V2 is freely accessible at http://www.zhounan.org/ferrdb/.

## INTRODUCTION

Ferroptosis is a form of iron-dependent cell death driven by the accumulation of toxic lipid reactive oxygen species, particularly lipid hydroperoxides (1). The mechanism of ferroptosis is complex and mainly related to metabolism, reactive oxygen species, and iron regulation (1). Emerging evidence proves that ferroptosis exhibits connections to numerous physiological activities such as tumor suppression, immune response, development, and senescence. Importantly, ferroptosis ubiquitously plays a key role in multifarious diseases. Ferroptosis is involved in tumorigenesis, progression, metastasis, and drug resistance in lung cancer, breast cancer, hepatocellular carcinoma, bladder cancer and so on (2–5). Ferroptosis also plays a critical role in non-neoplastic diseases including neurogenic disease (e.g. Alzheimer's disease, amyotrophic lateral sclerosis, and Parkinson's disease) (6–8), infectious diseases (9), autoimmune diseases (10), retinal diseases (11), tissue injuries (12) and some rare disorders (1). Collectively, ferroptosis is vitally implicated in a wide variety of diseases, as well as indicative for novel approaches of disease diagnosis, treatment, and prognosis prediction.

Owing to ferroptosis’ critical role in a broad set of biological contexts, the number of publications in this field continues increasing, from a few in 2012 when the term was coined to thousands nowadays. To assist ferroptosis research and knowledge sharing, we created and published the first version of FerrDb (FerrDb V1) in 2020 (13). As the first database dedicated to the ferroptosis field, FerrDb V1 has helped many researchers to obtain important discoveries. For example, Kishk *et al.* found ferroptosis as a candidate prognostic and target pathway for COVID-19 (14); Luo *et al.* discovered that ferroptosis impacts tumor immunity and it can help to improve the efficacy of patients’ immunotherapy (15); Zhao *et al.* revealed that gene regulators of ferroptosis play an essential role in osteosarcoma chemoresistance (16); He *et al.* constructed a ferroptosis score model and revealed the therapeutic liability of ferroptosis in melanoma (17); and Wu *et al.* constructed a ferroptosis-related signature to predict clinical outcomes and therapeutic responses in colon cancer patients (18). Overall, successful applications of FerrDb V1 in published studies clearly demonstrate its importance to ferroptosis research.

Ferroptosis is a research field that evolves rapidly, and many new articles have been published since the publication of FerrDb V1. To offer timely data and better service, here we update the database to a new version, FerrDb V2. We collected new articles and performed manual curation using a fine-tuned procedure from the one for FerrDb V1. We integrated gene-related data from external resources to add new features. We also re-designed the user interface to display content in a more user-friendly way.

## MATERIALS AND METHODS

### Article collection

We carried out search in the PubMed (https://www.ncbi.nlm.nih.gov/pubmed) database using ‘ferroptosis’ as the search term to collect ferroptosis-related journal articles. Because FerrDb V2 is an aggregate of intermediate minor updates, articles were searched at two time points. The first was on 21 April 2021 (for articles of year 2020), and the second was on 17 January 2022 (for articles of year 2021). The two-step search found 2504 articles in total, with 826 and 1678 articles from the first and second search, respectively. All found articles were interrogated in the present study.

### Data curation and annotation

To curate and annotate ferroptosis regulators and ferroptosis-disease associations from the collected articles, an adapted strategy from the one for FerrDb V1 was used in this study. The two strategies are quite similar, so we only describe the changes to FerrDb V2. (i) As shown in Table 1, there are seven curated data sets belonging to two primary and three secondary categories. (ii) At most time, evidence text without change was directly extracted from the original article; evidence text will be summarized from the source content if it is necessary, but this is a rare case. (iii) Ferroptosis markers do not form a unique primary category anymore, and they have been assigned into the regulator category. (iv) To annotate a gene as a ferroptosis marker, evidence from experimental research is mandatory. (v) A new data set, namely unclassified gene, has been added; when a gene lacks enough evidence to identify itself as neither a driver nor a suppressor, and when it also lacks supports to be a marker, then it will be annotated as an unclassified gene regulator.

**Table 1.** Annotation data in FerrDb V2

Data set	Primary category	Secondary category	Description
Driver	Regulator	Gene	A gene that promotes ferroptosis
Suppressor	Regulator	Gene	A gene that prevents ferroptosis
Marker	Regulator	Gene	A gene that indicates ferroptosis occurrence
Unclassified gene	Regulator	Gene	A gene that is associated with ferroptosis, but its regulatory role is unclear
Inducer	Regulator	Substance	A chemical entity that can cause ferroptosi
Inhibitor	Regulator	Substance	A chemical entity that can restrict ferroptosis
Ferroptosis-disease association	Association	Ferroptosis-disease association	Ferroptosis has two opposite effects on disease: aggravating an illness or alleviating an unpleasant situation

NB The table is adapted from the article of Zhou and Bao (13), with approval from the copyright holder.

For gene regulators, fundamental gene features (e.g. symbol, identifier and full name) were collected from the HGNC, Ensembl and UniProt databases (19–21). For substance regulators, basic information (e.g. identifier) was collected from the PubChem database (22).

### Integrating external gene resources

For genes with protein products, three-dimensional (3D) protein structures are from the AlphaFold Protein Structure Database (https://alphafold.ebi.ac.uk/) (23). Protein's 3D structure in that database is predicted by the AlphaFold system from its amino acid sequence and the prediction accuracy is regularly competitive with experiment (24).

Gene-related data in healthy tissues were downloaded from the Genotype-Tissue Expression (GTEx) Portal (https://gtexportal.org/home/). The GTEx project is an ongoing effort to study tissue-specific gene expression and regulation in samples collected from 54 non-diseased tissue sites across nearly 1000 individuals, primarily using molecular assays including WGS, WES and RNA-seq (25). For gene expression, RNA-seq TPM values in the V8 release were downloaded. For protein expression, the proteomics data of 32 normal human tissues from 14 individuals generated by TMT-MS3 based quantitative mass spectrometry was downloaded from the Enhancing GTEx (eGTEx) project (26,27). The data of tissue specific distribution of proteins was also downloaded from eGTEx.

Gene expression (RNA-seq TPM values) and gene-level copy number data in tumor cells generated by the Cancer Cell Line Encyclopedia (CCLE) project were downloaded from the 22Q2 public release in the DepMap Portal (https://depmap.org/portal/) (28). Gene effect scores and gene dependency probabilities derived from CRISPR knockout screens in tumor cells were downloaded from the same origin. The CRISPR data were generated by the Broad's Project Achilles (29,30). Protein abundance in cancer cells was downloaded from the study by Nusinow *et al.* (31).

Tumor patient-derived data were downloaded from the TCGA program in the GDC Data Portal (https://portal.gdc.cancer.gov/) (32). Gene expression levels, protein abundances, and matched clinical information of sample donors were collected. Protein expression levels were quantified with reverse phase protein array (RPPA) in MD Anderson Cancer Center (33). Because preprocessed TPM values are not available after last update of the GDC portal, raw RNA-seq read counts were downloaded and edgeR was then used to calculate TMM values from read counts (34). Differentially expressed genes (DEGs) between groups were analyzed using DESeq2 in R (35). DEGs were defined by fold change ≥2 and *P* < 0.05. For survival analysis, the mean or median gene expression levels were used to stratify cancer patients into two groups, namely low and high expression groups. The lifelines python library was used for survival analysis (http://lifelines.readthedocs.org/) (36,37).

Gene expression data in non-tumor diseases were downloaded from the Gene Expression Nebulas (GEN) database (https://ngdc.cncb.ac.cn/gen/) (38). One RNA-seq data set per disease was collected. In total, read counts and TPM values of 34 diseases were downloaded. Differentially expressed genes defined by fold change ≥2 and *P* < 0.05 were detected from read counts using DESeq2 in R (35).

CRISPR-based gene knockout and RNA interference (RNAi) are two widely used loss-of-function approaches to interrogate gene function in functional genomics. CRISPR gRNAs from the human CRISPR knockout pooled library GeCKO v2 was a gift from Feng Zhang (Addgene #1000000048, #1000000049) (39). The GeCKO library contains 123 411 gRNAs for 19 050 genes. Lentiviral short hairpin RNA (shRNA) libraries and their corresponding RNAi sequences targeting ferroptosis gene regulators were developed by Moffat and colleagues (40), and the data were collected from the GPP Web Portal (https://portals.broadinstitute.org/gpp/public/gene/search).

### Development and deployment

The front end of FerrDb V2 was developed with mainstream web developing techniques, such as HTML5, CSS3 and JavaScript. Web page layout was developed with Bootstrap v5 (https://getbootstrap.com/). The jQuery library was used to extend JavaScript programming (https://api.jquery.com/). DataTables was used to display data in interactive table on the web page (https://datatables.net/). The Plotly graphing library was used to plot interactive diagrams on the web page (https://plotly.com/). Cytoscape.js was used to visualize pathway and regulation network on the webpage (41). Interactive 3D protein structure is embedded on the web page using the service provided by Mol* viewer which automatically loads structure from the AlphaFold Protein Structure Database (42). Drug information is retrieved on request from the DrugBank database via UniProt's REST API (43).

The back end of FerrDb V2 was developed with the Python Django web framework (https://www.djangoproject.com/). Curated data were stored in an SQLite database (https://www.sqlite.org/). Gene-related data from external resources were saved in files in formats of csv, pickle, and feather. The GSEApy python package was used to perform gene set enrichment analysis (http://gseapy.rtfd.io/).

FerrDb V2 is deployed using Apache HTTP server in Ubuntu.

## RESULTS

### Core data

We collected 2504 ferroptosis articles published in year 2020 and 2021. Since 784 articles were already included in FerrDb V1, ferroptosis regulators and ferroptosis-disease associations in FerrDb V2 were manually curated from a total number of 3288 articles.

In FerrDb V2, the 621 ferroptosis gene regulators are from 564 unique genes (Table 2 and Supplementary Figure S1). There are 264 drivers, 238 suppressors, 9 markers and 110 unclassified genes. With respect to substances that regulate ferroptosis, there are 201 inducers and 179 inhibitors. FerrDb V2 also contains 143 ferroptosis-disease associations, with 90 exacerbating diseases and 53 alleviating illness conditions. In comparison, FerrDb V1 contains 108 drivers, 69 suppressors, 111 markers, 35 inducers, 41 inhibitors and 95 ferroptosis-disease associations.

**Table 2.** Statistics of data in FerrDb V2 compared with that in FerrDb V1

Data set	Unique counts		Total annotations	
	FerrDb V2	FerrDb V1	FerrDb V2	FerrDb V1
Gene	564	259	/	/
• Driver	264	108	369	150
• Suppressor	238	69	348	109
• Marker	9	111	11	123
• Unclassified gene	110	/	116	/
Substance	/	/	/	/
• Inducer	201	35	298	54
• Inhibitor	179	41	246	46
Ferroptosis-disease association	143	95	232	135
• Ferroptosis aggravates disease	90	49	128	58
• Ferroptosis alleviates disease	53	46	104	77

NB Unique counts are mainly used for result report. The unique gene count (564) is less than the sum of driver, suppressor, marker, and unclassified gene, because 46 genes are classified into multiple data sets, as shown in the Supplementary Figure S1.

In FerrDb V2, ferroptosis gene regulators are distributed across eight gene types (Table 3). There are 487 genes with protein product, 31 microRNAs, 18 circular RNAs, 17 long non-coding RNAs, 7 unknown, 2 pseudogenes, 1 small nucleolar RNA and 1 readthrough. The percentage of non-coding genes in FerrDb V2 is approximately 13.6%, compared to 7.3% in FerrDb V1.

**Table 3.** Gene type distribution in FerrDb V2 compared with that in FerrDb V1

Gene type	FerrDb V2 count	FerrDb V1 count
Gene with protein product	487	240
RNA, micro	31	9
circular RNA	18	NA
RNA, long non-coding	17	2
Unknown	7	6
Pseudogene	2	NA
Readthrough	1	1
RNA, small nucleolar	1	1

NB: Count is calculated based on unique gene in Table 2. The information about gene type is mainly from the HGNC database. In case of unavailable gene type from HGNC, the curator will define a suitable gene type, according to the curator's understanding of the gene.

### Database description

#### Overview

**Figure 1.**
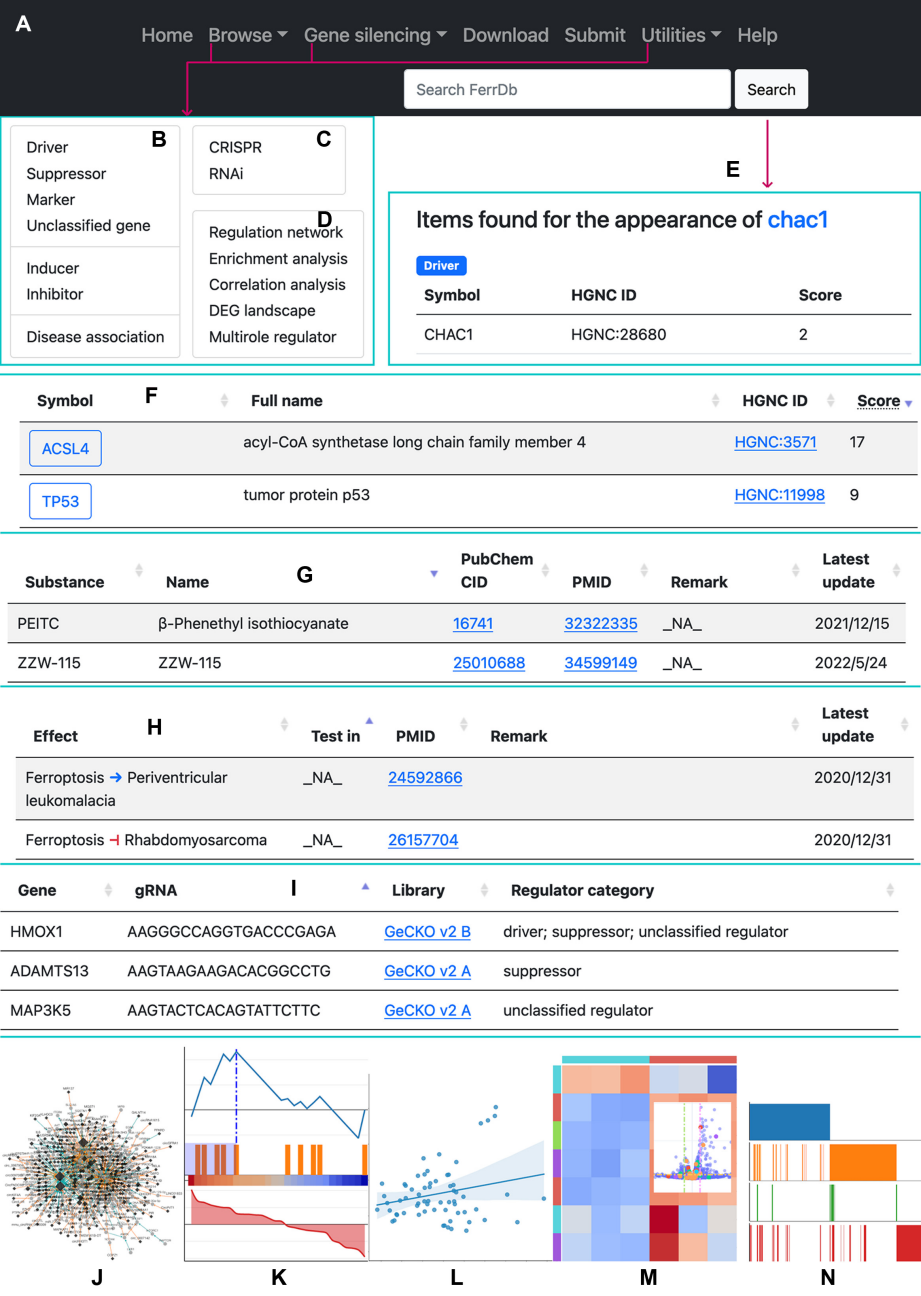
Overview of FerrDb V2. (**A**) The navigation bar. (**B**) The expanded ‘Browse’ drop-down menu. (**C**) The expanded ‘Gene silencing’ drop-down menu. (**D**) The expanded ‘Utilities’ drop-down menu. (**E**) An example of searching ‘chac1’. (**F**) An example of a table of ferroptosis drivers. (**G**) An example of a table of ferroptosis inducers. (**H**) An example of a table of ferroptosis-disease associations. (**I**) An example of a table of CRISPR gRNAs. (**J**) A schematic presentation of the ‘Regulation network’ utility. (**K**) A schematic presentation of the ‘Enrichment analysis’ utility. (**L**) A schematic presentation of the ‘Correlation analysis’ utility. (**M**) A schematic presentation of the ‘DEG landscape’ utility. (**N**) A schematic presentation of the ‘Multirole regulator’ utility.

In the navigation bar, links to resources in FerrDb V2 are provided (Figure 1A–D). The search box accepts user input and looks for content that contains the search text (Figure 1E). Core data in FerrDb V2 can be accessed via the ‘Browse’ drop-down menu in the navigation bar (Figure 1B).

Core data are divided into individual annotation data sets, namely driver, suppressor, marker, unclassified gene, inducer, inhibitor, and ferroptosis-disease association. Items of a data set are listed in a table on the web page. Driver, suppressor, marker, and unclassified gene are displayed in a similar manner, so a driver table is shown as an example (Figure 1F). In the table, genes are shown in rows, with columns showing HGNC symbol, full name, HGNC ID, and score. The score of a gene represents the number of publications where the gene has been studied. Ferroptosis inducers and inhibitors are shown in the same format, and a table of inducers is shown in Figure 1G as an example. The attributes shown in columns include substance label, name, PubChem identifier, PMID, remark and time of last update. On the webpage that shows ferroptosis-disease associations (Figure 1H), the ‘Effect’ column shows whether ferroptosis exacerbates or alleviates a disease.

On the ‘Gene silencing’ drop-down menu (Figure 1C), links to CRISPR and RNAi data sets are provided. These data are shown in table on the webpage (Figure 1I and Supplementary Figure S2), and they can be used to query gRNA and shRNA sequences of ferroptosis gene regulators.

FerrDb V2 also provides useful utilities. They are available on the ‘Utilities’ drop-down menu (Figure 1D). They can be used to inspect regulation network of ferroptosis gene regulators (Figure 1J), to perform gene set enrichment analysis (Figure 1K), to find gene correlations (Figure 1L), to examine the landscape of DEGs (Figure 1M), and to browse gene regulators with multiple roles (named as ‘multirole’ regulators) (Figure 1N).

#### Gene detail page

**Figure 2.**
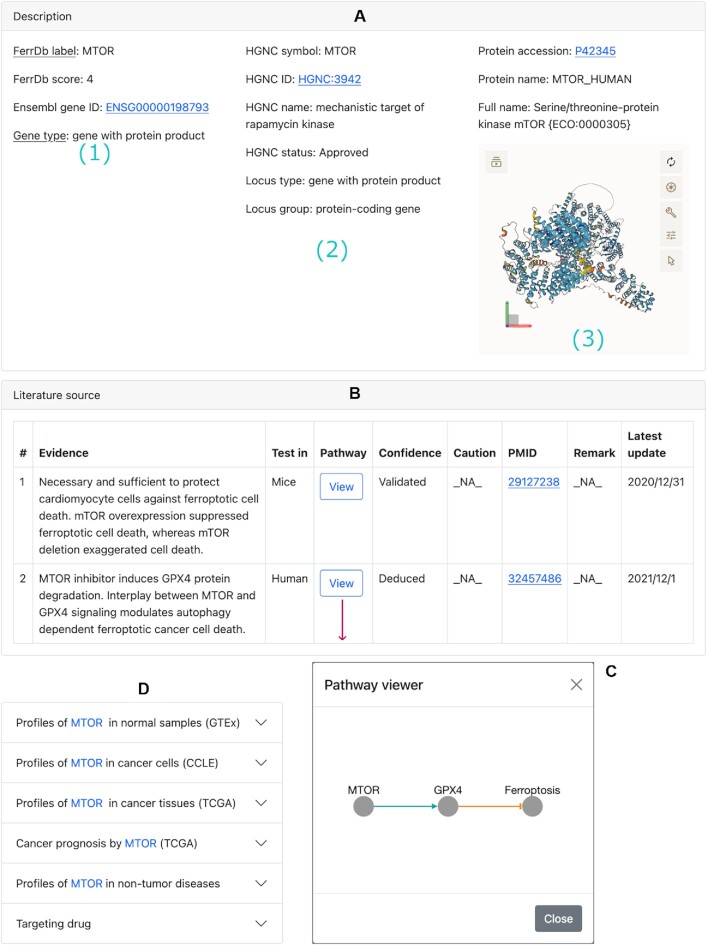
An example of gene detail page, with contents organized into sections. (**A**) The ‘Description’ section: panel 1 shows gene description from the curator; panel 2 shows information from the HGNC database; and panel 3 shows information from the UniProt database and 3D protein structure from the AlphaFold protein structure database. Panel 2 or 3 may not be always shown due to unavailable data from the external source. (**B**) The ‘Literature source’ section. (**C**) An example of a regulation pathway from (B). (**D**) A collection of collapsed sections to gene-related data from external resources.

Every ferroptosis gene regulator has a detail page. Clicking on the gene symbol (e.g. MTOR) in the table will open its detail page (Figure 2). Contents on this page are organized into sections. The ‘Description’ section shows basic information about this gene (Figure 2A). In the ‘Literature source’ section, study details about this gene are listed in a table (Figure 2B), and clicking on the ‘View’ button of an entry will display the pathway of how the gene regulates ferroptosis through interaction with other biomolecules (Figure 2C).

**Figure 3.**
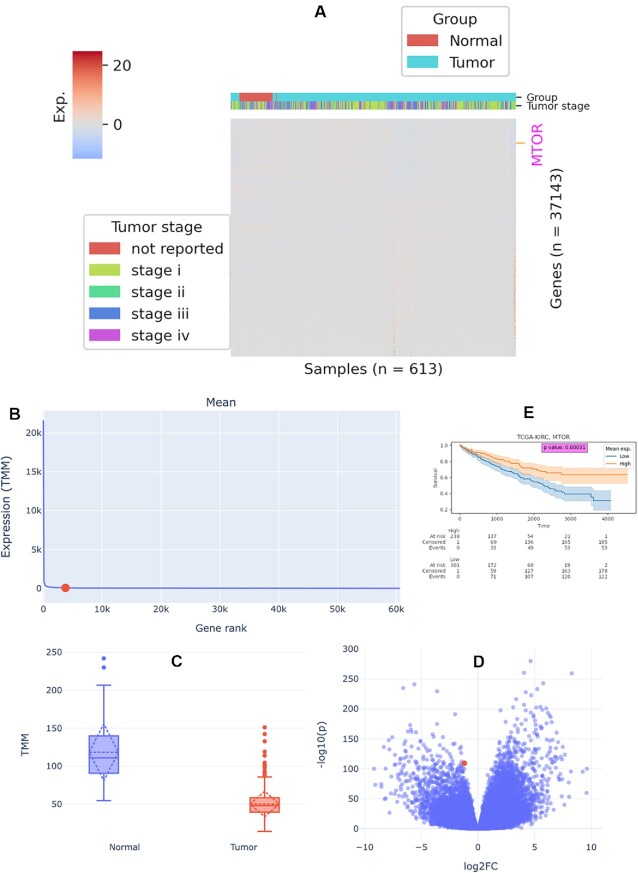
An example of TCGA profiles of MTOR. (**A**) RNA level of MTOR compared to other genes in the transcriptomic landscape of all samples in the TCGA-KIRC project. The expression level is represented as *z*-scored log_2_(TMM + 1), where TMM is the normalized read counts using edgeR TMM method. Genes on the row are not clustered but are sorted by expression sum in ascending order (bottom to top). Columns are clustered but the dendrogram is not shown. (**B**) Mean RNA level of MTOR compared to other genes in all samples in the TCGA-KIRC project. (**C**) Comparison of gene expression levels between normal and tumor groups in the TCGA-KIRC project. (**D**) Differential expression of MTOR (denoted by red point) between normal and tumor samples in the TCGA-KIRC project. (**E**) Prognosis of tumor donors stratified by mean MTOR RNA level in the TCGA-KIRC project.

Following the ‘Literature source’ section is a collection of sections that shows gene-related data from external resources (Figure 2D). In the ‘GTEx profile’ section, the RNA level, protein abundance, and tissue-specific protein expression of the gene in normal tissues in comparison with other genes are shown (Supplementary Figure S3). In the ‘CCLE profile’ section, the RNA level, protein abundance, gene-level copy number, and CRISPR-derived gene effect and gene dependency of the gene in cancer cells in comparison with other genes are shown (Supplementary Figure S4). In the ‘TCGA profile’ section, transcriptomic and proteomic profiles are available (Figure 3 and Supplementary Figure S5). For transcriptomic profiles, RNA level of the gene compared to other genes (Figure 3A, B and Supplementary Figure S5A) and differential gene expressions (Figure 3C, D) are shown. For the proteomic profile, protein abundance of the gene compared to other genes are shown (Supplementary Figure S5B). Since TCGA provides follow-up data of participants, survival analysis of cancer patients by the gene can be performed in the ‘Cancer prognosis’ section (Figure 3E and Supplementary Figure S5C). In the ‘Non-tumor disease profile’ section, RNA level of the gene compared to other genes and differential gene expressions are shown (Supplementary Figure S6). In the ‘Targeting drug’ section, known drugs targeting the gene are listed.

#### Utilities

**Figure 4.**
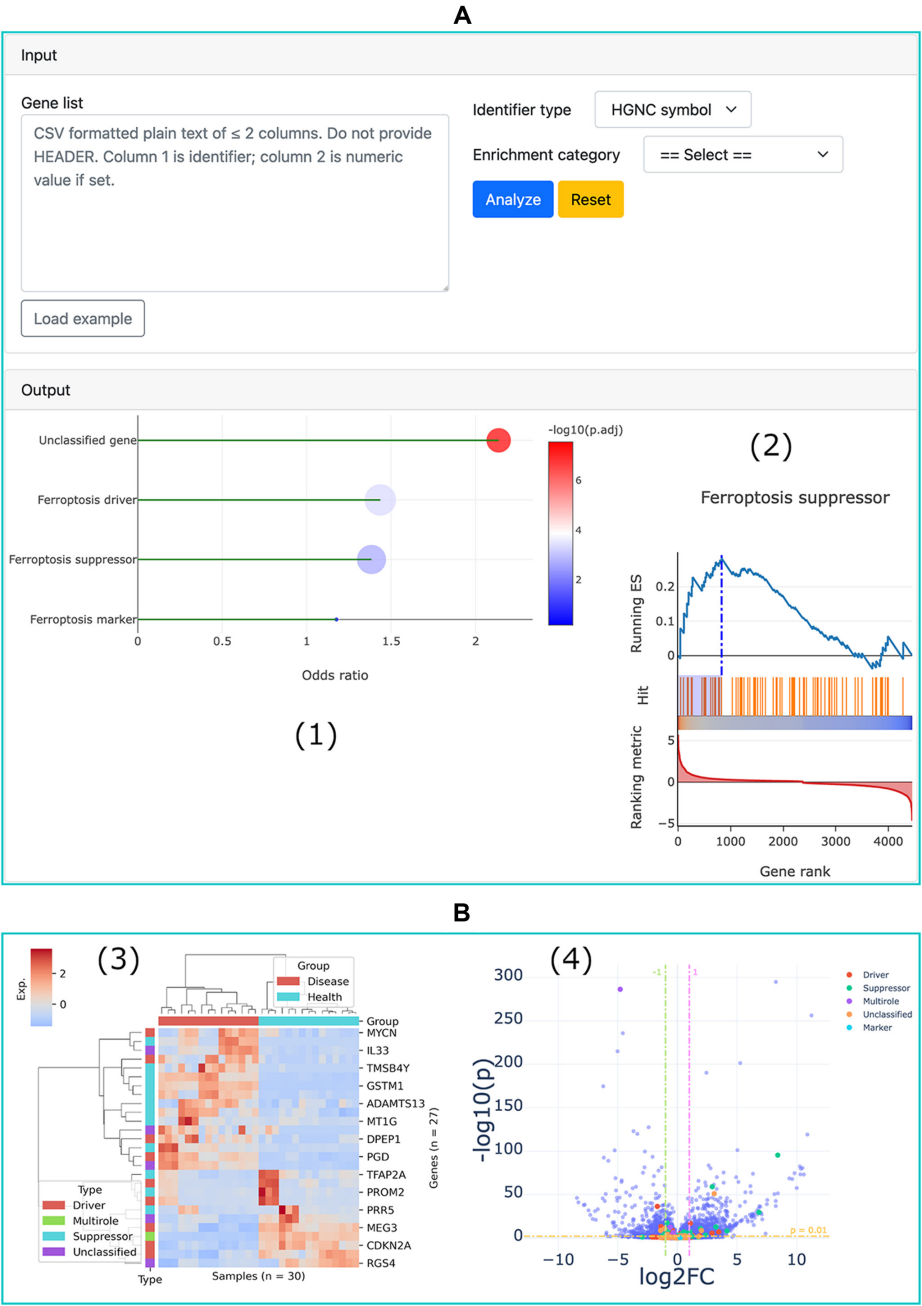
Enrichment analysis and DEG landscape utilities. (**A**) Input and output panels of the enrichment analysis utility: (1) an example of the result of over-representation analysis; and (2) an example of the result of GSEA analysis. (**B**) DEG landscape of the type 1 diabetes as an example: (3) hierarchical clustering of differentially expressed ferroptosis gene regulators, with values scaled by *z*-score and (4) differential expression of all genes between diabetes and healthy controls, with ferroptosis gene regulators encoded by different colors.

The ‘Regulation network’ utility can be used to browse the integrated regulation network of ferroptosis gene regulators. This function has been detailed in FerrDb V1, so we do not repeatedly introduce it here. The ‘Enrichment analysis’ utility can be used to test if a given list of genes is statistically enriched in any of the ferroptosis regulator set based on either over-representation or GSEA analysis (Figure 4A) (44,45). The ‘DEG landscape’ utility can be used to inspect the overall differential expressions of all ferroptosis gene regulators in a disease at once (Figure 4B). The ‘Correlation analysis’ utility can be used to analyze the correlation between two genes and that between two omics profiles of one gene (Supplementary Figure S7). The ‘Multirole regulator’ utility can be used to find genes that have multiple regulatory effects on ferroptosis (Supplementary Figure S8).

Download and upload functions are also available in FerrDb V2. The corresponding link in the navigation bar can be used to download data from or upload data to FerrDb V2.

### Application case

**Figure 5.**
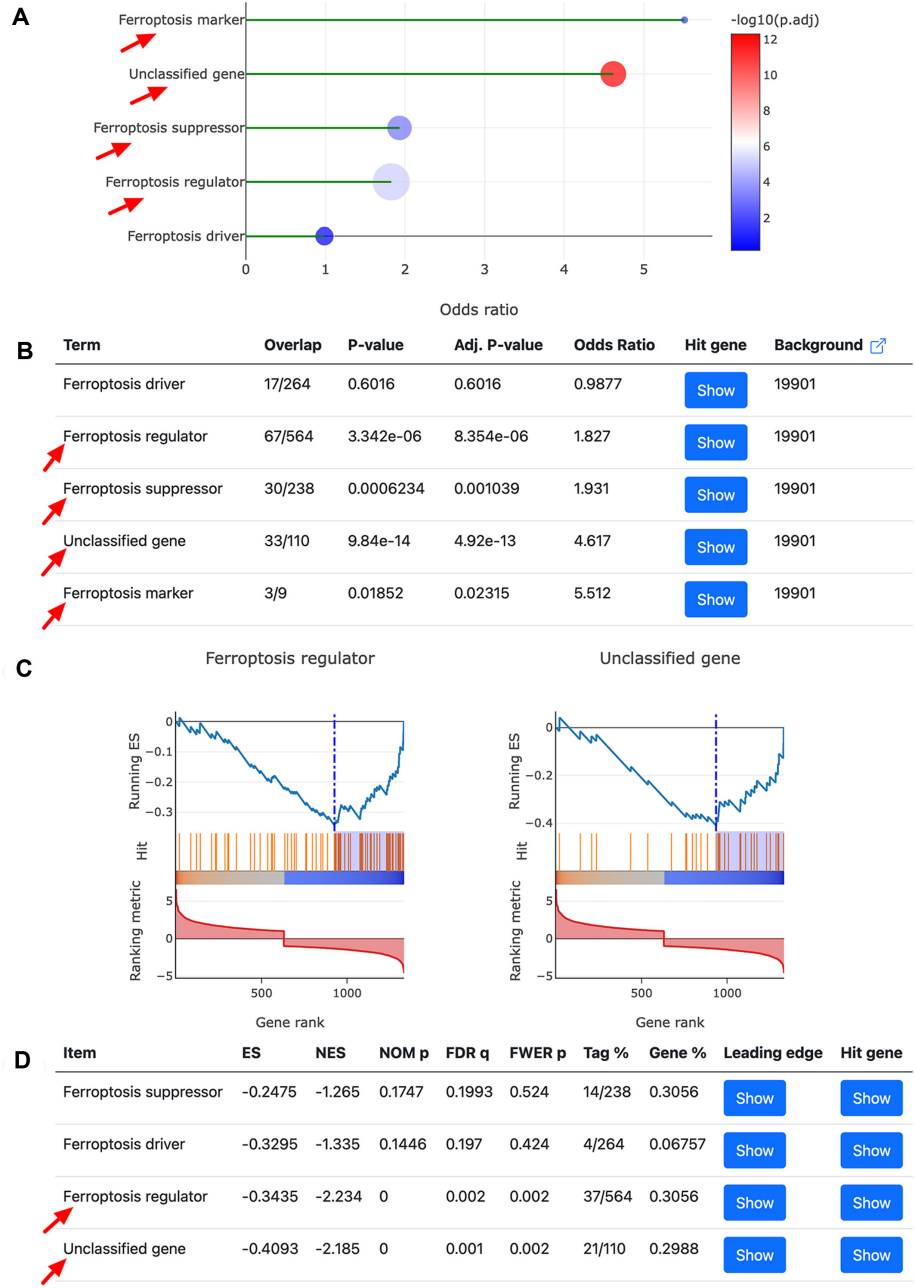
Enrichment results of OA DEGs by the ‘Enrichment analysis’ utility in FerrDb V2, with significantly enriched terms indicated by red arrows. Here the term ferroptosis regulator denotes a merged gene set of driver, suppressor, marker, and unclassified gene. (**A** and **B**) Results of over-representation analysis. (**C** and **D**) Results of GSEA analysis.

Here we demonstrate how the newly added ‘Enrichment analysis’ utility in FerrDb V2 can be used to assist users in their research. In a study carried out by Fisch *et al.*, they identified 1332 differentially expressed genes (DEGs) between normal and osteoarthritis (OA) articular cartilage (46). Miao *et al.* themselves built a set of ferroptosis gene regulators which contains 67 drivers and 50 suppressors, and finally they confirmed that ferroptosis was involved in OA progression and that ferroptosis blockade may serve as an alternative therapeutic strategy for OA treatment (47). We downloaded those OA DEGs, sorted them by log2 fold change, and then input them into the ‘Gene list’ text area in the input panel (Figure 4A). We performed both over-representation and GSEA analyses. As can be seen from Figure 5, the results from our analysis also indicate that OA DEGs are significantly enriched in ferroptosis. What's more, Miao *et al.* found that OA DEGs were enriched in the ferroptosis suppressor set but not the driver set (47), and our analysis yielded the same result (Figure 5A, B). It should be emphasized that Miao et al. did not use our data in their analysis, but we achieved consistent findings. Therefore, this example of application clearly demonstrates FerrDb V2’s convenience, capability, and reliability.

## DISCUSSION

Thanks to the rapid progress in the ferroptosis field during the last 2 years, upgrading FerrDb V1 is urgent and necessary. Ferroptosis marker formed a distinct category called ‘marker’ in FerrDb V1, but it belongs to the regulator category in FerrDb V2. This change has split the old marker data into two parts: the marker and unclassified gene sets. During the curation process, we noticed that marker genes usually come with the function to regulate ferroptosis with underlying mechanisms known or not. It is practically normal that when a gene is known to regulate ferroptosis then changes to this gene can indicate the occurrence of ferroptosis under certain circumstances. Conversely, if a gene is firstly identified as a ferroptosis marker, it will be reasonable to assume that the gene can regulate ferroptosis to some extent. Annotation data sets in FerrDb V2 also show this trend. For eight of the nine marker genes, three (TF, TFRC and CHAC1) are also drivers, and five (GPX4, HSPB1, NFE2L2, FTH1 and SLC40A1) are also suppressors.

In FerrDb V2, a gene associated with ferroptosis but without known regulatory effect is annotated as an unclassified gene regulator. This kind of gene was called ferroptosis marker in FerrDb V1. We noticed it was hard to assign some gene into any category when developing FerrDb V1, but we just simply put them into the marker category. This would cause confusions sometimes. It is necessary to make ferroptosis marker explicit. Therefore, a new date set, unclassified gene, was added to FerrDb V2. Now, a gene is identified as a marker only when experimental evidence is available. This adjustment improves data quality and will help to precisely interpret gene's function.

The constituent types of gene regulators have big difference between FerrDb V1 and FerrDb V2. Although the largest proportion of genes are protein-coding, there are about 6% more non-coding genes in FerrDb V2 than in FerrDb V1. In addition, circular RNAs and pseudogenes that are not in FerrDb V1 are included in FerrDb V2. The increase in gene types implies the complexity of ferroptosis regulation and further studies are required for deeper understanding of ferroptosis mechanisms.

In FerrDb V2, gene-related data from external resources have been integrated. This enriches the content on the gene detail page. For example, RNA-seq data and clinical information from TCGA make it possible and convenient to predict cancer prognosis. The external data have also helped us to develop new utilities, such as the ‘DEG landscape’ which can be used to interrogate differential gene expression patterns of ferroptosis gene regulators in cancers and non-tumor diseases.

The new ‘Enrichment analysis’ utility is another fantastic and important function. It can be used to test if a target gene list is enriched in any set of the ferroptosis gene regulators. Even though there are many web-based and stand-alone tools for gene set enrichment analysis, they do not have ferroptosis regulator data and cannot perform such analysis. The data is unique here, and currently the analysis is only available in FerrDb V2.

In Figure 4B, we can see that 27 ferroptosis gene regulators are differentially expressed in the RNA-seq data of type 1 diabetes. Interestingly, these genes clearly and correctly separate type 1 diabetes and healthy controls, implying their involvement in the development of the disease. This hints that further study of ferroptosis in the pathology of diabetes is worthwhile.

In conclusion, here we have updated FerrDb V2 from the first version. It has more data, functions, and conveniences. It can be used as not only a ferroptosis resource, but also an integrated analysis platform. It will be regularly updated to support long-term service. It is expected to help more researchers and propel advancement in the ferroptosis field.

## DATA AVAILABILITY

FerrDb V2 is freely accessible at http://www.zhounan.org/ferrdb/.

## ACCESSION NUMBERS

Non-tumor RNA-seq data accession numbers in GEN are: GEND000001, GEND000002, GEND000005, GEND000009, GEND000014, GEND000016, GEND000033, GEND000036, GEND000069, GEND000070, GEND000072, GEND000084, GEND000085, GEND000086, GEND000087, GEND000174, GEND000179, GEND000180, GEND000181, GEND000212, GEND000329, GEND000333, GEND000334 and GEND000339.

## Supplementary Material

gkac935_Supplemental_File
