# Supplementary material for: FerrDb V2: update of the manually curated database of ferroptosis regulators and ferroptosis-disease associations
Source: Nucleic Acids Res. 2022 Oct 28;51(D1):D571–82. doi: 10.1093/nar/gkac935 (PMC9825716; doi:10.1093/nar/gkac935)
Supplement: gkac935_Supplemental_File [file gkac935_supplemental_file.pdf]

Supplementary Figure and legends

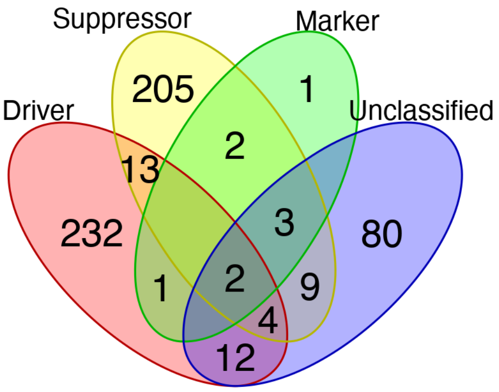

Supplementary Figure 1. Overlaps between ferroptosis gene regulators.

(1)

Gene Symbol	Clone ID	Legacy Clone Name	Target Seq	Vector
ABCC1	TRCN0000059363	NM_004996.2-1128s1c1	CCTCTCTGTTTAAGGTGTTAT	pLKO.1

(2)

Matching Transcripts for Gene	Match Regions	Match %	SDR Match %	Intrinsic Score	Adjusted Score	Matches Other Gene in Same Taxon?	Orig. Target Gene ID
XM_011522497.1, NM_004996.4, XM_017023243.2, XM_017023242.1, XM_017023239.1, XM_017023241.1, XM_017023237.1, XM_011522498.2, XM_017023238.1, XM_017023240.1	CDS	100	100	13.2	18.48	N	4363

(3)

Orig. Target Gene Symbol	Forward Oligo Sequence
ABCC1	CCGGCCTCTCTGTTTAAGGTGTTATCTCGAGATAACACCTTAAACAGAGAGGTTTTTG

(4)

Reverse Oligo Sequence	Regulator category
AATTCAAAAACCTCTCTGTTTAAGGTGTTATCTCGAGATAACACCTTAAACAGAGAGG	driver

Supplementary Figure 2. An example of RNAi data. The table is too wide, so columns are split into four parts, denoted by 1-4 in the diagram.

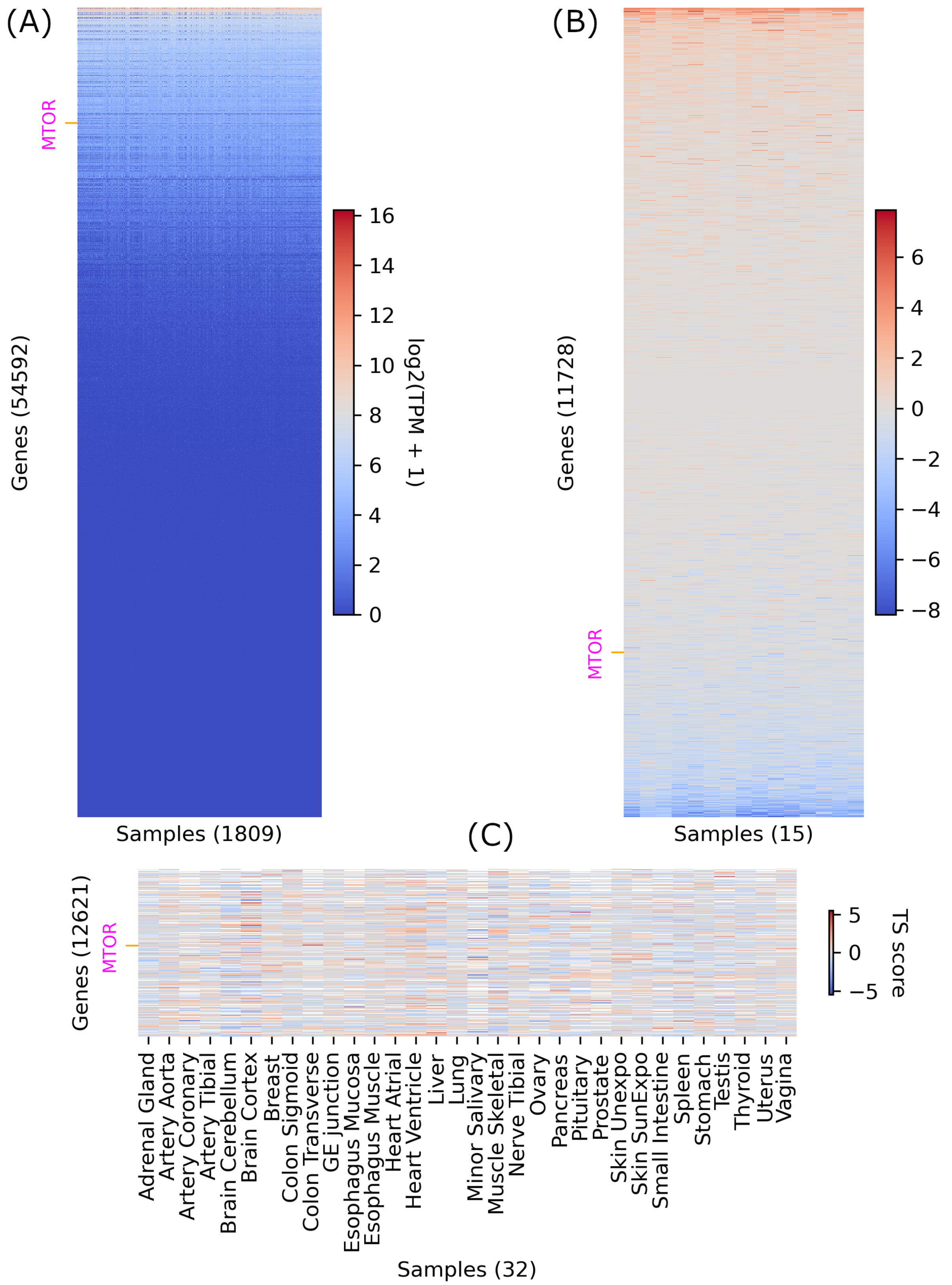

Supplementary Figure 3. An example of GTEx profiles of MTOR. (A) RNA level of MTOR compared to other genes in the transcriptomic panorama of skin samples. Values are represented as log2 transformed TPM, using a pseudo-value of 1. Rows are sorted by expression sum in ascending order (bottom to top); columns are sorted by expression sum in descending order (left to right). (B) Protein abundance of MTOR compared to other genes in the proteomic landscape of skin samples. Represented values are log2 transformed

relative protein abundances. Rows are sorted by abundance sum in ascending order (bottom to top). Columns are sorted by abundance sum in descending order (left to right).
(C) Tissue-specific (TS) protein expression of MTOR compared to other genes. Represented values are z-scored TS score. Rows are sorted by gene symbol in ascending order (bottom to top).

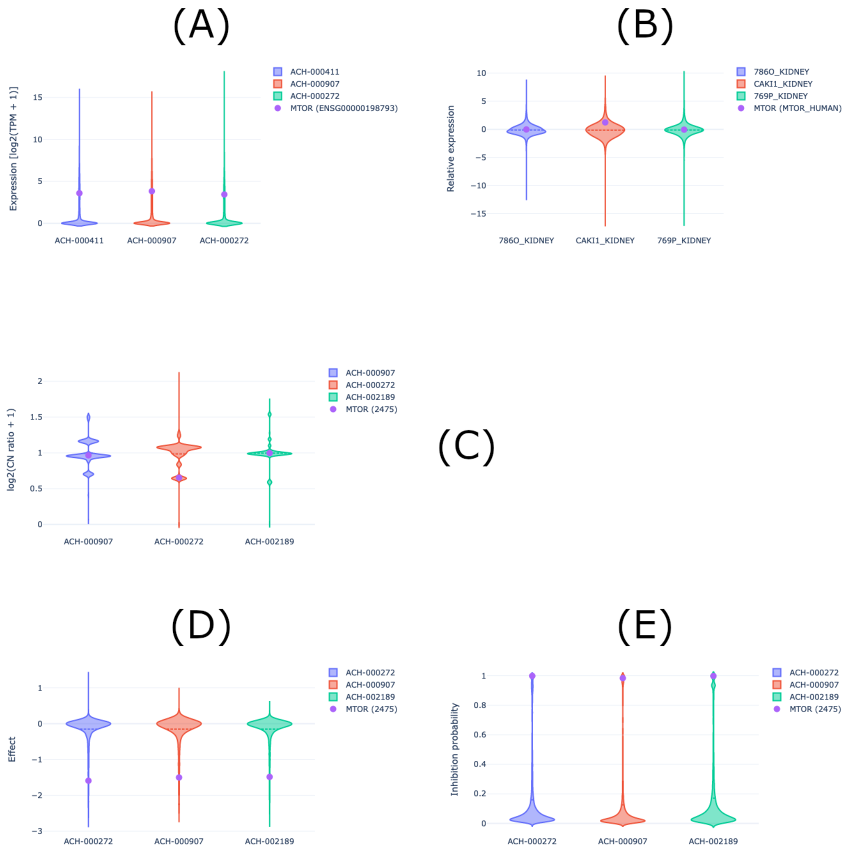

Supplementary Figure 4. An example of CCLE profiles of MTOR in clear cell renal carcinoma. (A) RNA level of MTOR compared to other genes in three cells. Values are represented as log2 transformed TPM, using a pseudo-count of 1, i.e., log2(TPM+1). (B) Protein abundance of MTOR compared to other genes in three cells. (C) Gene-level copy number of MTOR compared to other genes in three cells. The copy number value is log2 transformed with a pseudo-count of 1, i.e., log2(relative to ploidy + 1). (D) CRISPR knockout effect of MTOR compared to other genes in three cells. Negative scores imply cell growth inhibition and/or death following gene knockout. Scores are normalized such that nonessential genes have a median score of 0 and independently identified common essentials have a median score of -1. (E) CRISPR gene dependency probability (GDP) of MTOR compared to other genes in three cells. The GDP represents the likelihood that knockout of the gene has a cell growth inhibition or death effect.

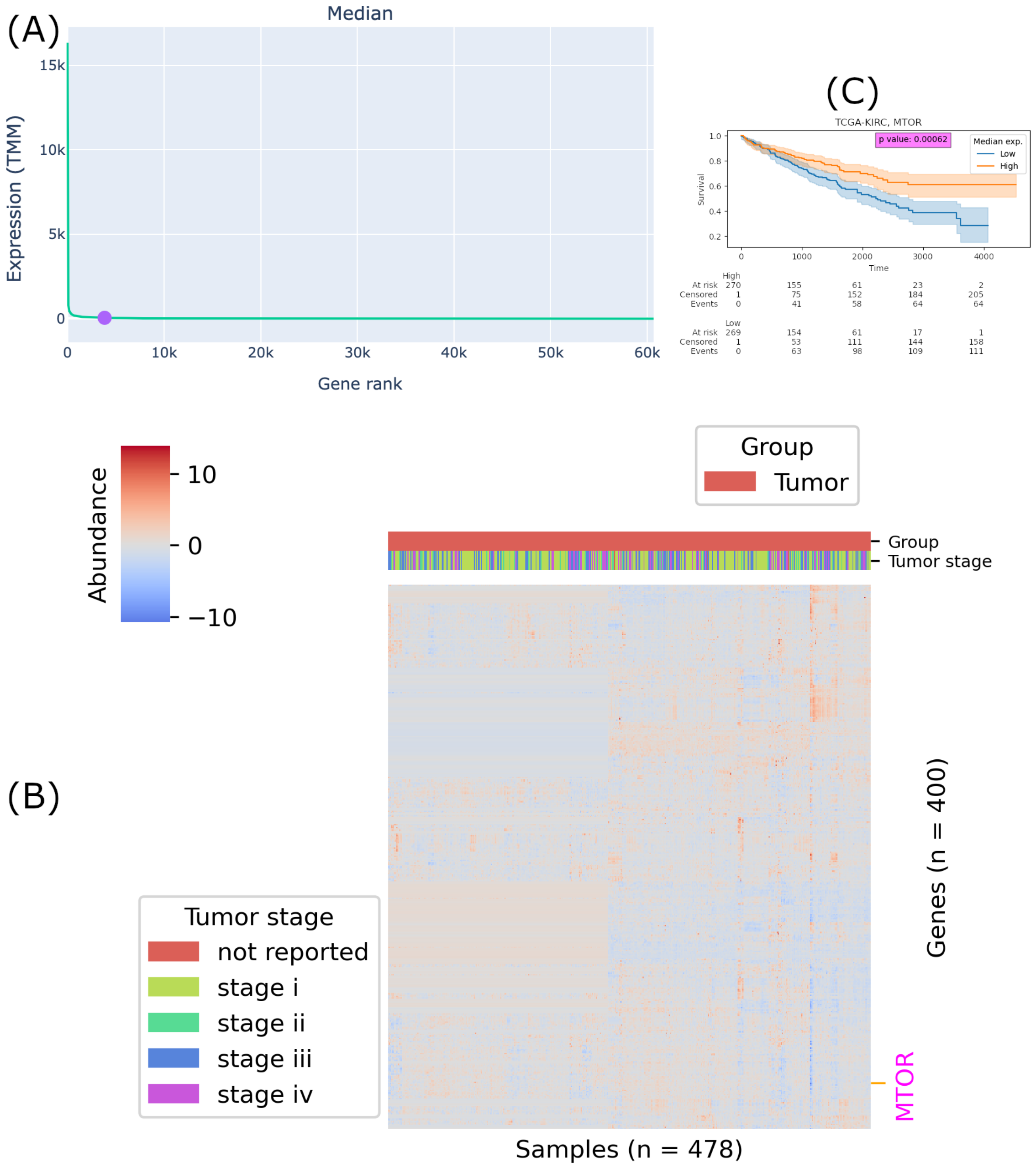

Supplementary Figure 5. An example of additional TCGA profiles of MTOR. (A) Median RNA level of MTOR compared to other genes in all samples in the TCGA-KIRC project. (B) Protein abundance of MTOR compared to other genes in the proteomic landscape of samples in the TCGA-KIRC project; rows and columns are clustered but the dendrograms are not shown. (C) Prognosis of tumor donors stratified by median MTOR RNA level in the TCGA-KIRC project.

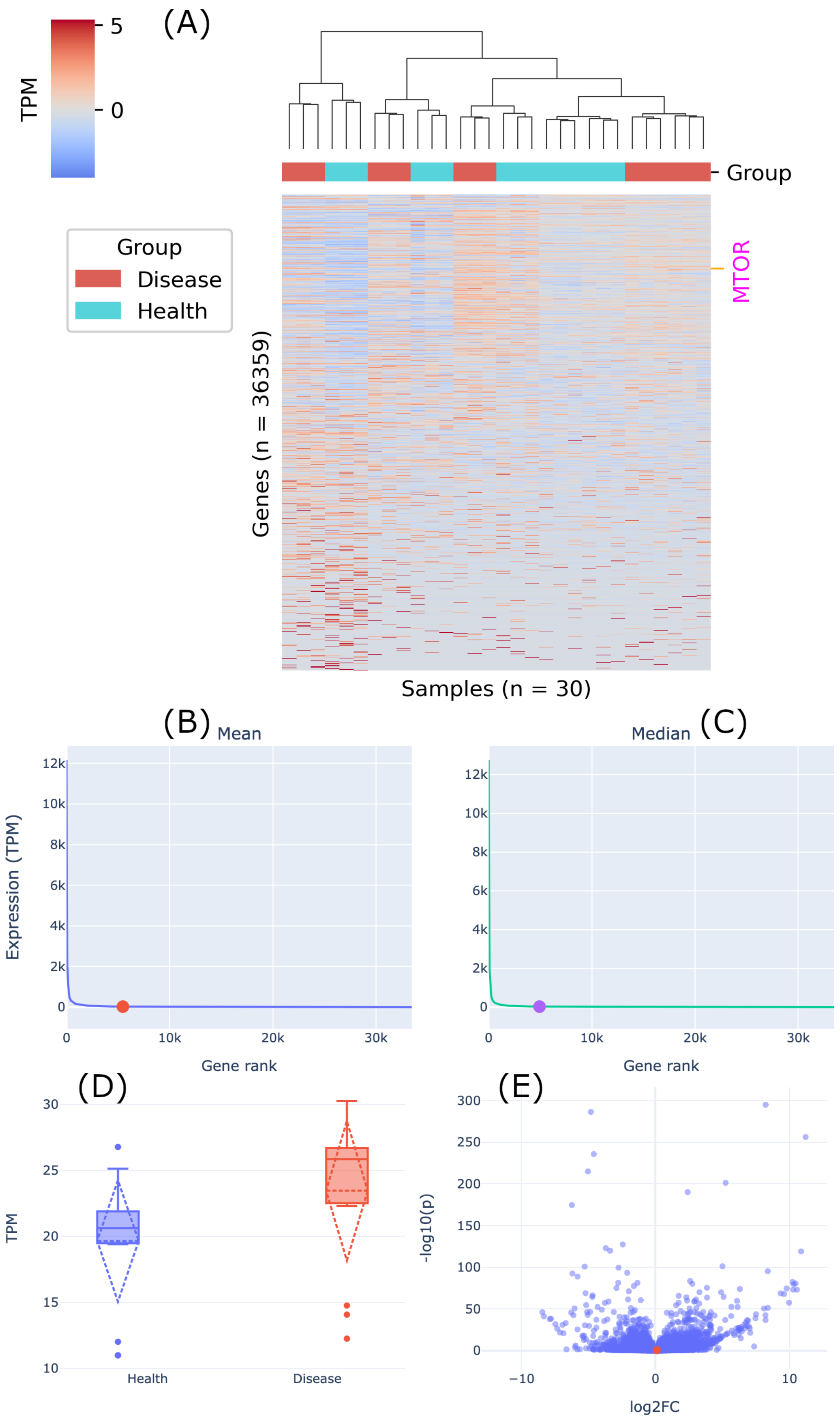

Supplementary Figure 6. An example of non-tumor profiles of MTOR. (A) RNA level of MTOR compared to other genes in the transcriptomic landscape of type 1 diabetes. Values are represented as z-scored TPM. Rows are not clustered but are sorted by expression sum in

ascending order (bottom to top). (B, C) Mean and median RNA levels of MTOR compared to other genes in type 1 diabetes. (D) Comparison of gene expression levels between type 1 diabetes and healthy controls. (E) Differential expression of MTOR between type 1 diabetes and healthy controls.

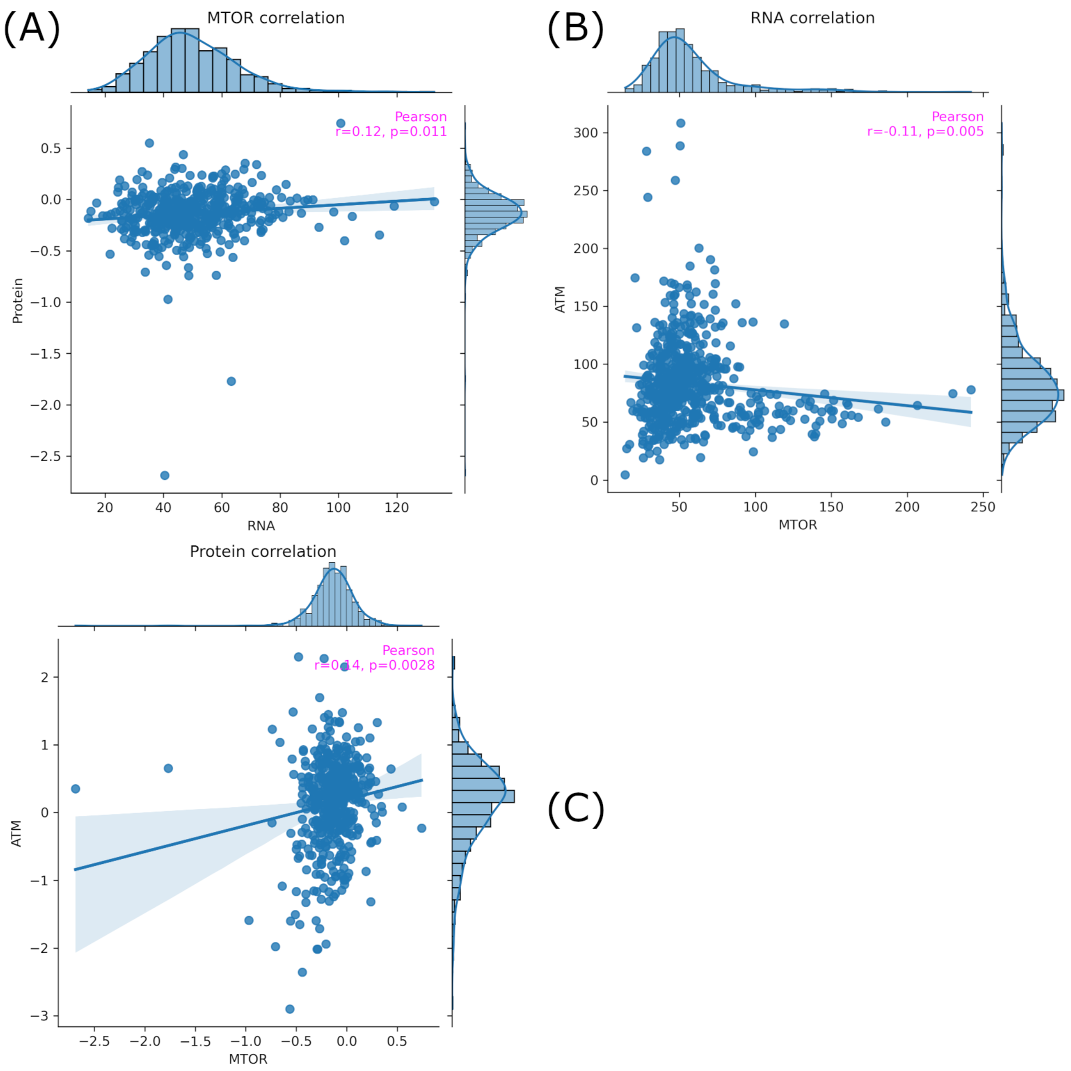

Supplementary Figure 7. An example of gene correlation analyses in the TCGA-KIRC project. (A) Correlation between the RNA and protein levels of MTOR. (B) Correlation between the RNA levels of MTOR and ATM. (C) Correlation between the protein levels of MTOR and ATM.

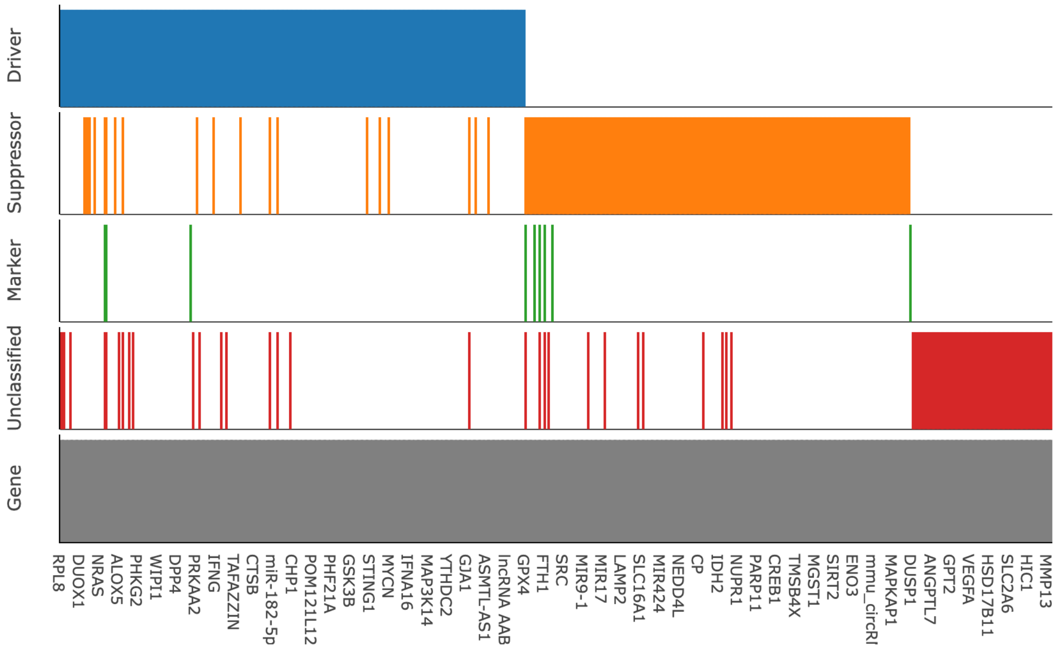

Supplementary Figure 8. Multirole ferroptosis gene regulator browser.
